# Engineering of small-molecule lipidic prodrugs as novel nanomedicines for enhanced drug delivery

**DOI:** 10.1186/s12951-022-01257-4

**Published:** 2022-01-24

**Authors:** Lingling Huang, Jianmiao Yang, Tiantian Wang, Jianqing Gao, Donghang Xu

**Affiliations:** 1 Department of Pharmacy, The Second Affiliated Hospital, Zhejiang University School of Medicine, Hangzhou, 310009 People’s Republic of China; 2 Zhejiang Province Key Laboratory of Anti-Cancer Drug Research, College of Pharmaceutical Sciences, Zhejiang University, Hangzhou, 310058 People’s Republic of China; 3 Cancer Center of Zhejiang University, Zhejiang University, Hangzhou, 310058 People’s Republic of China; 4 Taizhou Hospital of Zhejiang Province, Zhejiang, 317000 Taizhou People’s Republic of China

**Keywords:** Cancer, Small molecule, Lipidic prodrug, Nanomedicine, Drug delivery

## Abstract

A widely established prodrug strategy can effectively optimize the unappealing properties of therapeutic agents in cancer treatment. Among them, lipidic prodrugs extremely uplift the physicochemical properties, site-specificity, and antitumor activities of therapeutic agents while reducing systemic toxicity. Although great perspectives have been summarized in the progress of prodrug-based nanoplatforms, no attention has been paid to emphasizing the rational design of small-molecule lipidic prodrugs (SLPs). With the aim of outlining the prospect of the SLPs approach, the review will first provide an overview of conjugation strategies that are amenable to SLPs fabrication. Then, the rational design of SLPs in response to the physiological barriers of chemotherapeutic agents is highlighted. Finally, their biomedical applications are also emphasized with special functions, followed by a brief introduction of the promising opportunities and potential challenges of SLPs-based drug delivery systems (DDSs) in clinical application.

**Graphical Abstract:**

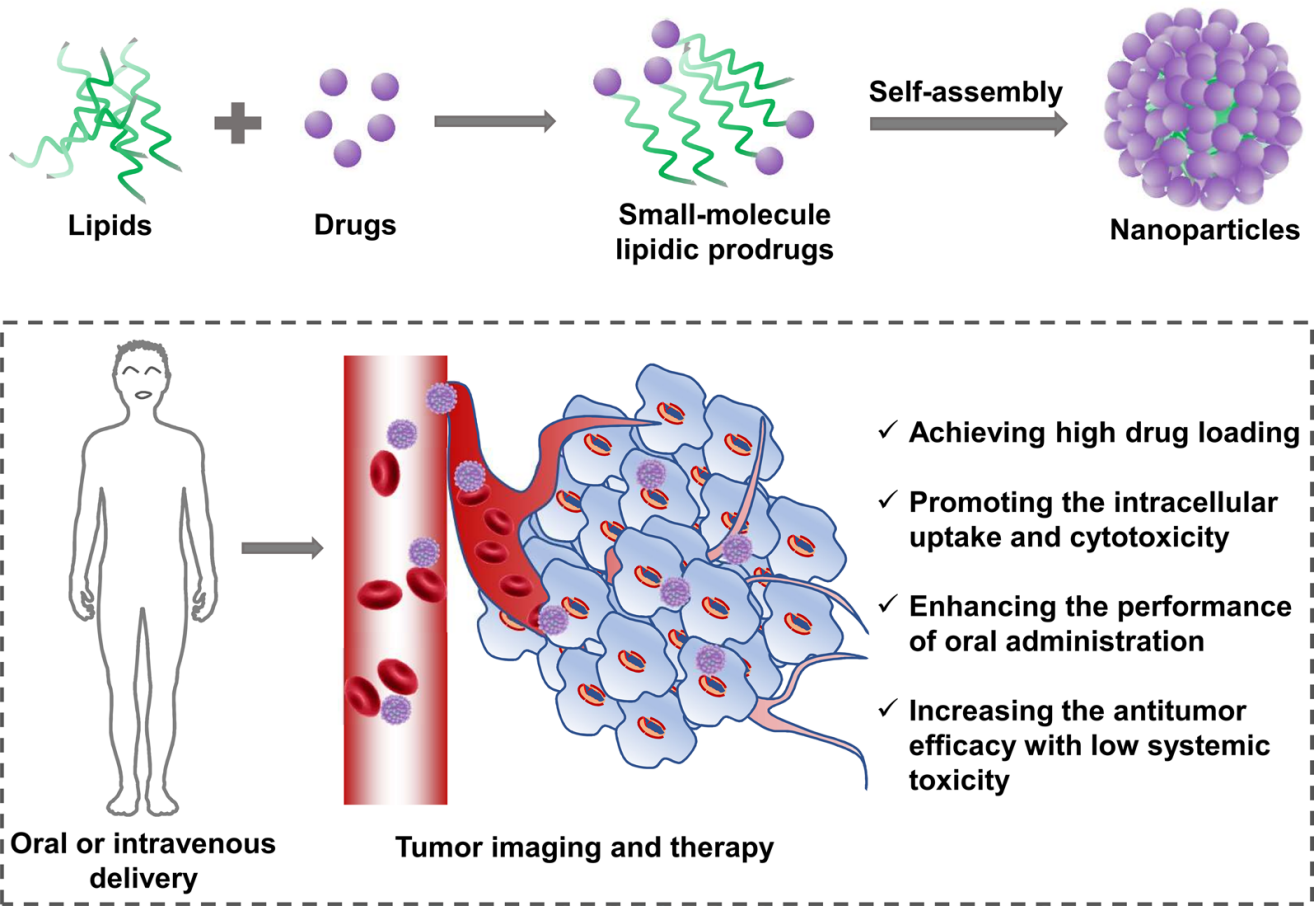

## Introduction

Cancer, a serious public health problem, poses a predominant threat to human life due to its high mortality [1]. Despite tremendous efforts and significant advances against cancer, treatment outcomes continue to be limited and far from satisfactory [2, 3]. Among the three major cancer therapeutic approaches (e.g., surgery, chemotherapy, and radiotherapy) in clinics, chemotherapy plays a pivotal role in the treatment of various tumors [4]. However, contemporary chemotherapeutic agents, mainly delivered through intravenous administration, suffer from several undesirable limitations, including (i) uncontrollable distribution at tumor lesions and therefore exerting cytotoxicity in normal cells; (ii) poor membrane permeability and retention through passive diffusion; and (iii) possible multidrug resistance induced by frequent drug administration, which extensively displays a narrow therapeutic window with reduced efficiency [5, 6]. To address these obstacles, numerous strategies have been developed to fight the anticancer war. Drug molecules physically packaged into nanocarriers are considered a promising approach in recent dosage development [7–11]. Unfortunately, inevitable toxic effects, such as nephrotoxicity of platinum drugs or cardiotoxicity of doxorubicin (DOX) formulations, are ascribed to drug leakage and burst release following in vivo circulation [12–14]. Additionally, as the preferred choice for encapsulation of poorly soluble drugs, the addition of extra surfactants, solubilizers, or large amounts of amphiphilic materials in drug formulations can inevitably increase the risk of hyposensitivity and immunogenicity [15]. To overcome the aforementioned drawbacks, there is an urgent need for sustained and controlled release properties of chemotherapeutic agents for specific tumor ablation and systemic toxicity reduction during drug delivery.

Prodrugs, referred to as inactive pharmacological molecules, have built-in structural lability by modification, that permits bioconversion in vivo through the enzymatic or chemical process of releasing various bioactive agents [16]. Since the prodrug strategy was initially recognized by Adrian Albert in 1958, it has provided various possibilities in the chemical design of existing cytotoxic drugs using biocompatible materials such as peptides, lipids, or amphipathic polymers [17–21]. As expected, prodrug strategy, as an increasingly versatile tool, is being used to address multiple barriers, including physicochemical, pharmacokinetic, or pharmacodynamics deficiencies of active agents that limit formulation inventions and generate uninspired biopharmaceutical performance [22–24]. Among them, stimuli-responsive prodrugs are more effective and desirable to realize accurate on-demand drug release and have been reported currently from various perspectives [25–28]. In recent years, amphiphilic polymers-based prodrugs have been widely investigated due to their improved drug solubility, chemical stability, and pharmacokinetic profile. Unfortunately, intrinsic flaws to the preparation of amphiphilic polymers still remain, including cumbersome and complex synthetic procedures, insufficient batch-to-batch reproducibility as well as potential systemic toxicity [29, 30]. These barriers limit the further clinical translation of polymer-drug conjugates. Compared with traditional polymer-based prodrugs, naturally abundant and commercially available small-molecule lipids (e.g., fatty acids, cholesterol, and glycerides) with attractive novel properties such as nontoxicity, biocompatibility, and biodegradability are ideal for the design of nanocarriers. These associated properties further provide an explanation of why a variety of excellent lipid-based nanocarriers are intensively employed for drug delivery [11, 31, 32]. As previously reported, fatty acids are involved in an active recycling process within biomembranes and further modulate the expression of pro-inflammatory cytokines and promote cell proliferation [33]. In most cases, small-molecule lipidic prodrugs (SLPs) have the capability to self-assemble into stable nanostructures without the support of additional amphiphilic excipients, which allows them to achieve high-efficiency drug loading over polymer or liposome-based encapsulation strategies [34–36]. Squalenoic acid (SQ) is widely exploited, for instance, by successful conjugation to a series of chemotherapeutic agents (e.g., gemcitabine (GEM) [37], paclitaxel (PTX) [38], DOX [39], and adenosine [40]). Encouragingly, more and more small-molecule lipids, especially unsaturated fatty acids, are used for SLPs-based self-assembly. The SLPs strategy allows us to achieve several advantages, including (i) minimal toxicity and negligible immunogenicity by using endogenous fatty materials; (ii) high membrane affinity, for instance, by facile membranes anchoring and enhanced cellular uptake; (iii) sustained drug release mediated by enzymatic or specific chemical hydrolysis; (iv) an improved pharmacokinetic profile that facilitates lipidic prodrugs reabsorbed across the tubular epithelium; and (v) bypassing hepatic first-pass effect and improving oral bioavailability via physiological lipid metabolic pathways [35, 36]. In this regard, it is noteworthy to mention that several promising SLPs have entered clinical trials, such as PTX-docosahexaenoic acid (PTX-DHA) [41], GEM-elaidic acid [42] and cytarabine-elaidic acid [43]. In detail, PTX-DHA versus dacarbazine was in phase 3 study for the treatment of metastatic malignant melanoma. In addition, when the SLPs-based nanoplatforms are used, elevated stability, targeting ability, or specific imaging can be further achieved by the additional decoration of nanocarriers with functional moieties.

## Conjugation strategies of SLPs

### Drug conjugates with fatty acids

**Fig. 1:**
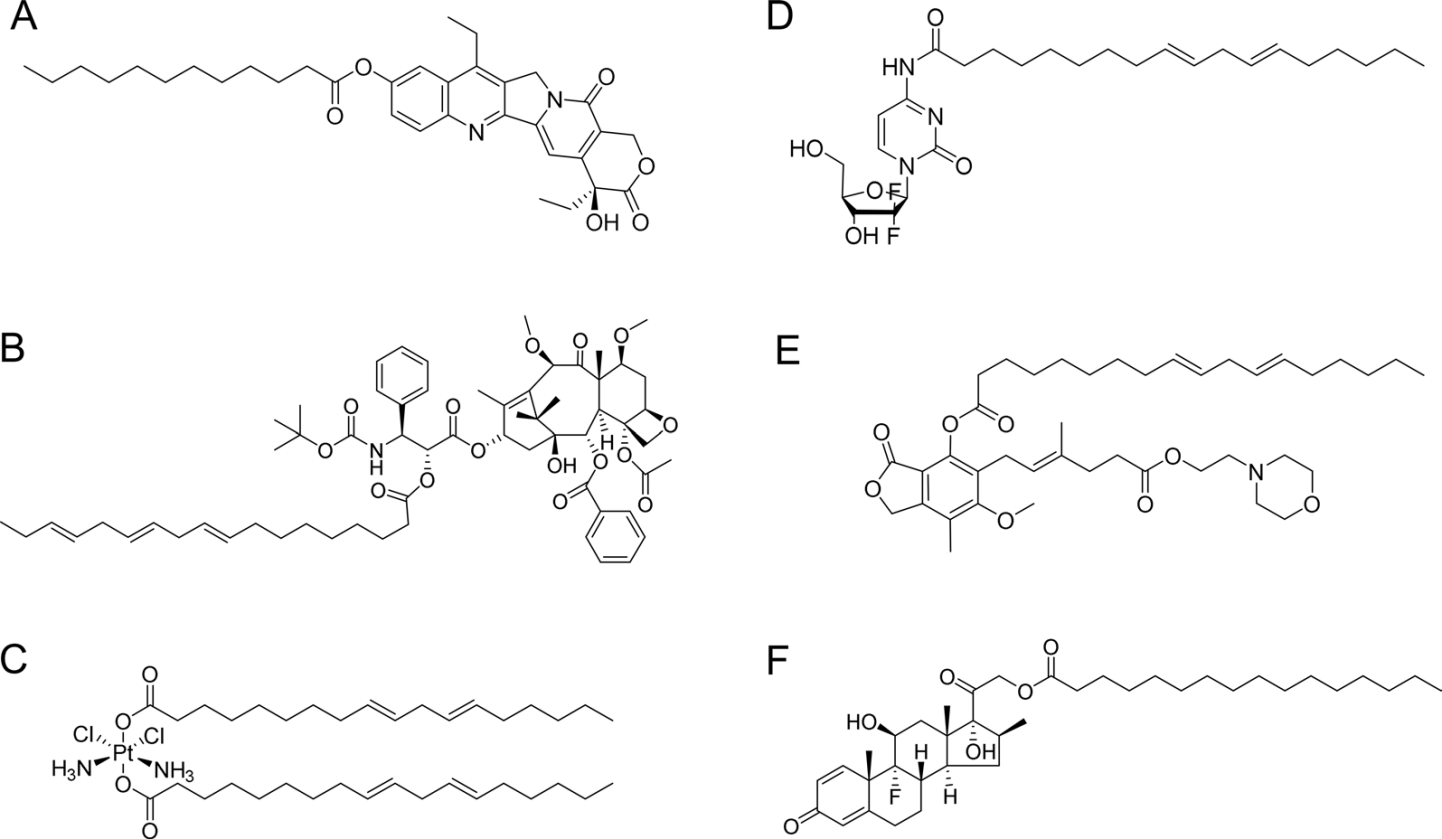
Fatty acids-based prodrugs of **A** SN38, **B** cabazitaxel, **C** cisplatin, **D** gemcitabine, **E** mycophenolate mofetil and **F** dexamethasone

**Fig. 2:**
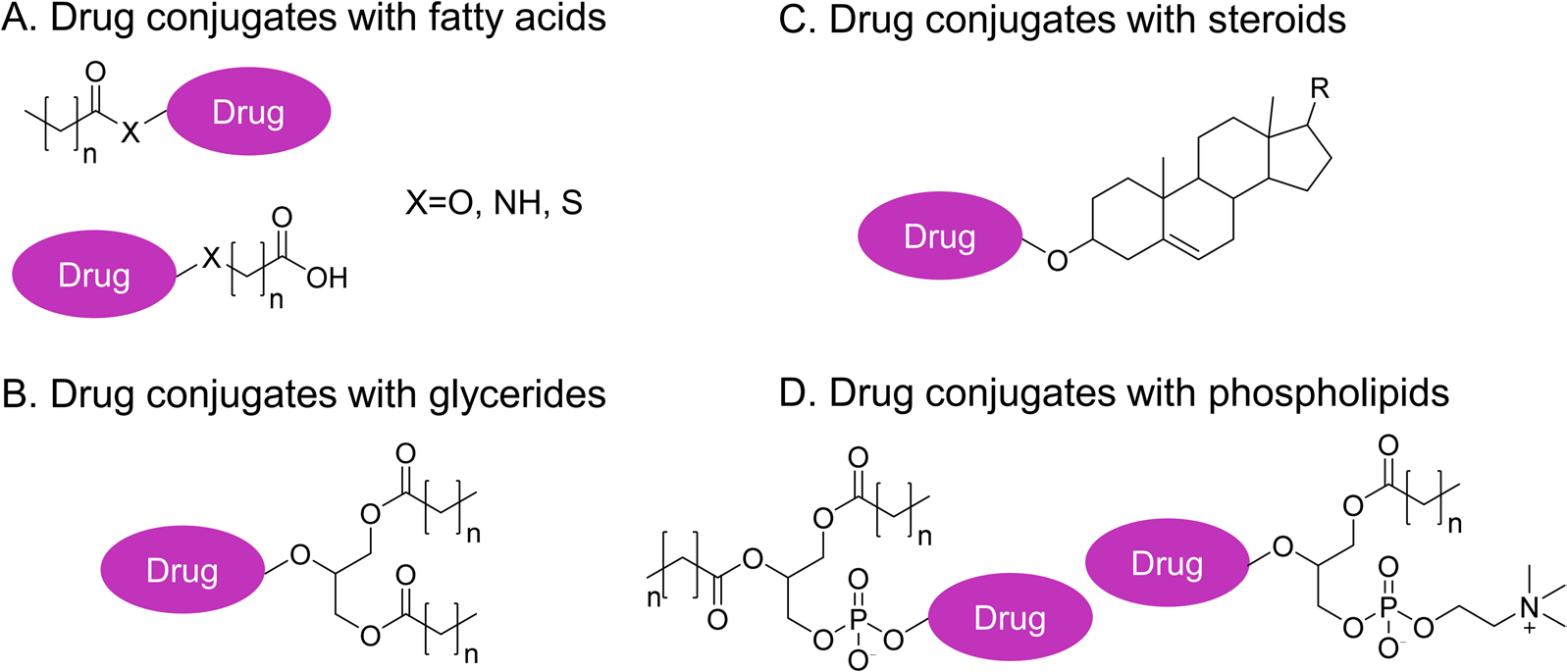
Conjugation strategies for lipidic prodrugs. **A** Drugs conjugated with fatty acids at the carboxylic acid end or the ω-carbon. **B** Drugs conjugated to glycerides at the sn2 position. **C** Drugs conjugated with steroids at the hydroxyl group of the steroidal ring. **D** Drugs conjugated to phospholipids via the sn2 hydroxyl group or via phosphate group

To synthesize lipidic prodrugs, fatty acids with a long hydrocarbon chain and free carboxylic acid are commonly used to bridge the free hydroxyl or amine functional groups of active drug molecules (Figs. 1 and 2) [44–47]. Fatty acids (e.g., SQ, DHA, and linoleic acid) and their derivatives can improve the drug penetration and uptake due to the lipid cell membranes. Meanwhile, the fatty acid chains facilitate the self-assembly process to form nanoparticles. As a result, for efficient treatment, numerous chemotherapeutic agents-fatty acid prodrugs have been developed [48–50]. 7-ethyl-10-hydroxycamptothecin (SN38) is an analog of camptothecin (CPT) with extraordinary antitumor activity [51]. Unfortunately, it is incompatible with conventional polymeric materials for drug delivery. In recent years, Wang’s group developed a variety of SN38-fatty acid conjugates that displayed an exceptional self-assembly capability in aqueous solutions [52, 53]. Interestingly, in comparison with free CPT-11, the generated supramolecular nanoassemblies demonstrated enhanced antitumor activity. Recently, a novel light-responsive nanoparticle based on fatty acid prodrugs was reported for spatiotemporally selective delivery of SN38 and photosensitizers [54].

### Drug conjugates with glycerides

Triglycerides (TG), as major constituents of dietary fat, are formed by bridging glycerol and three fatty acids via an ester linkage (Fig. 2). Carbons of acylated glycerol, usually at position 2, have been used for drug conjugation based on the glyceride template to mimic the TG metabolism pathway. Unlike most small-molecule drugs that undergo the first-pass effect, TG is firstly digested into diglyceride and ultimately into monoglyceride (MG) and fatty acids (FA) by the pancreatic lipase. Once absorbed into enterocytes, they get reacylated into TG and assembled into lymph lipoproteins, which are then accumulated into the lymphatic circulation. Therefore, TG-mimetic prodrugs with increased lipophilicity have the advantage of association with lipoproteins to facilitate the lymphatic transport pathway [55–57].

Recently, to enhance oral bioavailability, Porter’s research group reported a glyceride-mimetic prodrug of testosterone (TST) incorporated self-immolative (SI) spacers, which avoided substantial first-pass metabolism compared to the commercial drug testosterone undecanoate. To generate a TG-mimetic prodrug, TST was bridged with glyceride moiety via a C5 linker at the 2-position, in which a SI spacer reacted with the TST OH at its functional carboxyl end as well as inserted into the C5 linker to activate drug release [58]. Additionally, a combination of the βMe-branched linker to the glyceride ester could stabilize the enzymatic lability against gastrointestinal (GI) hydrolysis. In the GI tract, TG-mimetic prodrugs were converted into MG-like intermediates, absorbed, and reacylated into TG derivatives in the enterocytes. After being assembled into lipoprotein, it is subsequently distributed to the lymphatic transport pathway. Additionally, a glyceride-mimetic prodrug of docetaxel (DTX) with a reduction-sensitive disulfide bond has been implemented to promote oral absorption via a lymph transport mechanism and achieve on-demand drug activation in tumor tissue [59].

### Drug conjugates with steroids

Bile acids are important metabolites derived from cholesterol and can be delivered through bile acid transporters (Fig. 2). Due to the scavenging ability and anti-inflammatory effect of potent reactive oxygen species (ROS), the engineering of bile acid-derived prodrugs has been explored for the improvement in oral bioavailability and antitumor activities [60, 61]. Among various bile acid-based prodrugs in the current study, cholic acid-tethered tamoxifen with amine headgroup (CA-Tam_3_-Am) presented superior antitumor suppression. In addition, biophysical studies demonstrated that the amine group in tamoxifen molecules could stabilize the membrane binding [62]. Similarly, Sun’s research group designed a series of bile acid-based drug conjugation. An in vitro study showed that ursodeoxycholic acid-cytarabine conjugate displayed a remarkable antiproliferative effect compared to cytarabine in HepG2 cells, resulting from higher intracellular drug entrapment due to increased lipid properties. Besides, increased metabolic stability of ursodeoxycholic acid-cytarabine prolonged the in vivo circulation time, thereby providing cytarabine with a novel potential effect for the treatment of hepatocellular carcinoma [63].

Cholesterol is another steroidal derivative that is utilized for drug modification. As an indispensable substance of the cell membrane, drugs conjugated with cholesterol exhibit enhanced endocytosis. To endow the liposomes with indoximod (IND), an IDO-1 inhibitor, a cholesteryl-IND prodrug was synthesized for effective IND loading into the lipid bilayer. Combined with the remote incorporation of mitoxantrone into the liposome, the dual delivery carrier presented the feasibility of synergistic efficacy [64]. Furthermore, due to overexpressed low-density-lipoprotein receptors in tumor cells, cholesterol prodrugs could facilitate the process of cellular uptake. For instance, cholesterol-conjugated 5-fluorouracil outperformed free 5-fluorouracil in antitumor activity [65].

### Drug conjugates with phospholipids

Phosphorylcholine is a zwitterionic lipid component present at the outer surfaces of cell membranes that provides equal positive and negative charges for maintaining electrical neutrality (Fig. 2). The main strategy for forming phospholipid–drug conjugates (PDCs) is drug linkage at the phosphate moieties or glycerol backbone [66–68]. PCDs-based nanocarriers, especially prodrugs encapsulated in liposomes, show increased drug loading efficiency and enhanced stability and targeting ability than conventional liposomes in drug delivery due to increasing incorporation efficiency and overcoming transport resistant barriers [68–70]. Lanza’s group reported a lipase-labile prodrug in which hydrophobic drug DTX was coupled to the sn-2 acyl position of phosphatidylcholine. They proposed that fabricated αvβ3-Dxtl-PD NP failed to liberate PTX during circulatory transit, subsequently targeting the cell membrane for enzymatically triggered drug release [71]. In addition, phospholipid–doxorubicin conjugate (UnPC-hyd-DOX) exhibited ultrahigh drug loading content (56.2%) and facilitated cell internalization [72]. Besides, porphyrin-phospholipid conjugate-based nanovesicles realized significant photoacoustic and ROS generation capabilities, resulting in well-suited properties required for in vivo imaging as well as specific phototherapy [73–76].

## The advantages of SLPs-based drug delivery

### Achieving high drug loading in drug delivery systems (DDSs)

In preclinical studies, most hydrophilic drugs suffer from formidable challenges such as unsatisfactory drug leakage after systemic administration and low drug loading capacity [77–79]. Tethered lipid chains play an essential role in executing the enhancement of biocompatibility as well as the superior affinity between drugs and carriers, thus reducing drug leakage and potential systemic toxicity. For example, the DTX prodrug was prepared by conjugating DTX with oleic acid (OA) via a self-immolation thioether linkage. Notably, the carrier-free nanosystem presented a promising perspective for therapeutic application due to its high drug loading capacity (about 58%), redox-sensitivity to tumor cells, and long blood circulation duration [80]. Another interesting study reported that a linoleic acid–PTX conjugate with carrier-free characteristics displayed a novel self-assembly capability in aqueous solutions. Additionally, the nanoassemblies had good dispersibility and remarkable drug loading capacity [81]. Recently, Sauraj et al. designed a self-assembly nanocarrier for the effective delivery of 5-fluorouracil (5-FU) based on xylan-stearic acid conjugates. Upon lipid modification, the drug loading capacity of 5-FUSA significantly increased. Then, in vitro cell cytotoxicity determined that in comparison to free drugs, Xyl-SA/5-FUSA NPs exhibited higher cellular apoptosis against human colorectal cancer cells [82]. Moreover, adenosine, an endogenous immunomodulator, conjugating to squalene and then encapsulating *α*-tocopherol resulted in high drug loading and biocompatible. Selectively delivering adenosine and antioxidants with SQ-based nanoparticles could serve as a safe approach for mitigation of acute inflammation in rodents [83].

### Promoting the intracellular uptake and cytotoxicity of therapeutic agents

A chemotherapeutic agent, with a lipid moiety, results in higher lipophilicity and metabolic stability of the parent drug, which can facilitate cellular uptake via passive diffusion, particularly in cases when there is overexpression of low-density lipoprotein (LDL) receptors on tumor cells. Among them, polyunsaturated fatty acids (PUFAs), the important biomembrane components composed of multidouble bonds in hydrocarbon chains, play a significant role in signaling processes as well as regulating the dynamics and fluidity of cell membranes [35]. It is noteworthy to emphasize that a variety of PUFAs-drug analogs have been confirmed with prominent cellular uptake activity [34]. Additionally, diverse cytotoxic drug molecules chemically tethered with PUFAs provide a promising therapeutic platform.

**Fig. 3:**
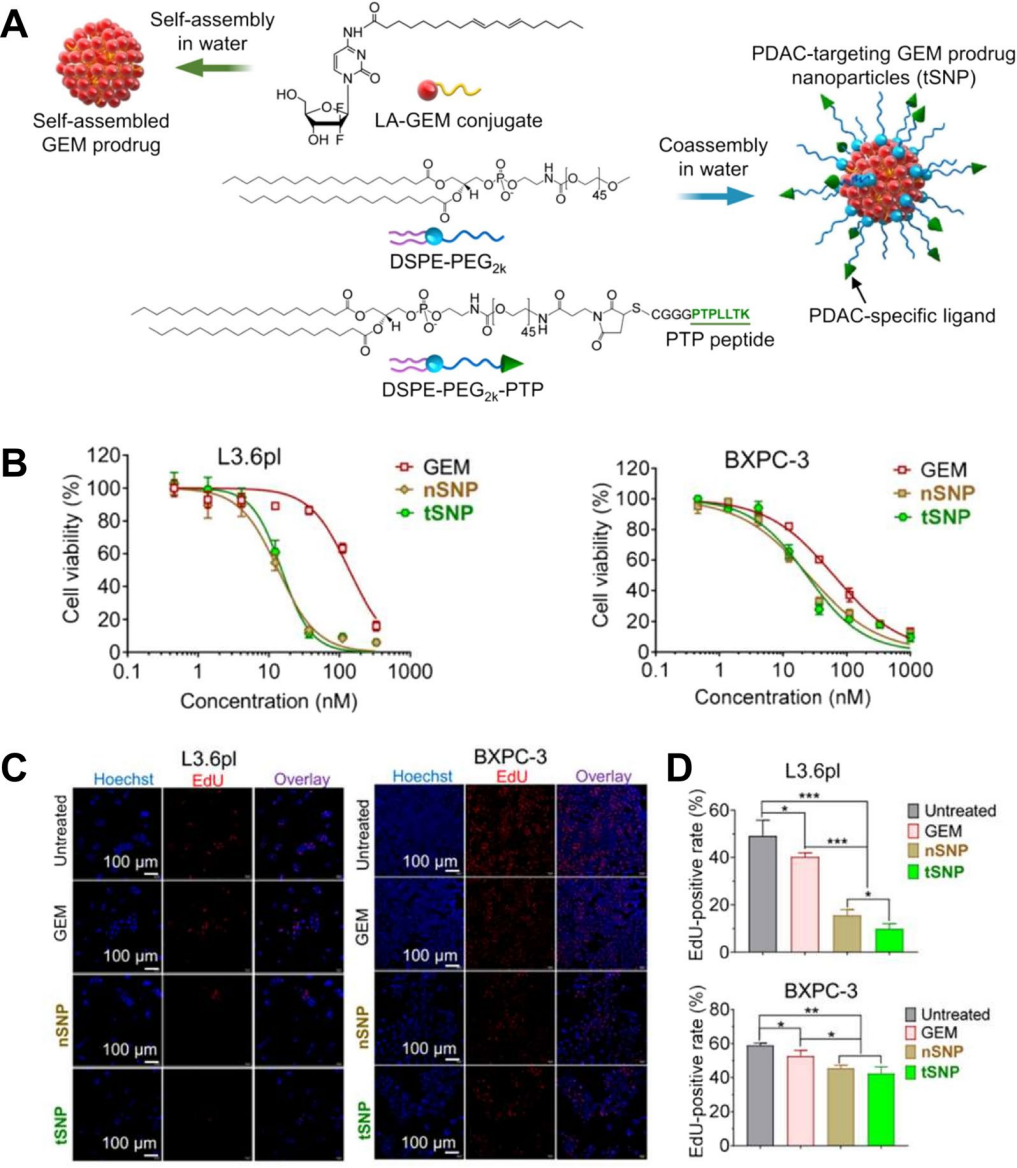
**A** Schematic diagram depicting the self-assembling process of GEM-prodrug-based nanoparticles. **B** In vitro cytotoxicity of nSNP and tSNP in L3.6pl and BXPC-3 cells examined by the cell counting kit-8 assay. **C** and **D** Click-iT EdU assay for analyzing the cell proliferation after different treatments. (Reprinted from Ref. [50] with permission. Copyright 2020, American Chemical Society Ltd)

GEM, an analog of deoxycytosine, can be rapidly incorporated into replicating DNA for termination of chain elongation, subsequently inducing cell death. As an efficacious chemotherapy available for various solid tumors, it is limited by serious intrinsic obstacles, including short circulation half-life due to metabolic instability, poor diffusion into tumor cells owing to the development of drug resistance, and its hydrophilic moieties. Hence, to address these defects of GEM, Wang’s group described a PUFAylation approach that attaches GEM to linoleic acid (LA) via an amide linkage (Fig. 3) [50]. In vitro release kinetics illustrated that LA-GEM nanoparticles could be considered as a reservoir to impede burst drug release, ultimately protecting GEM from rapid deactivation by cytidine deaminase. Moreover, use of this strategy made the GEM prodrug sufficiently amphiphilic and feasibly increased its in vitro cytotoxicity in L3.6pl and BXPC-3 cells compared to free GEM. Interestingly, LA-GEM nanoparticles unequivocally changed the transport pattern of free GEM depending on the membrane nucleoside transporter proteins, evidenced by the limited reduction of cytotoxicity after coincubation with nucleoside transporter inhibitors. Additionally, surface decoration of prodrug nanoparticles with a tumor-specific peptide ligand further exhibited synergistic antitumor activity in two preclinical xenograft models. Besides, a palmityl-tethered GEM derivative in thermosensitive hydrogel was designed as a long-acting drug delivery platform to achieve metabolic stability and improve intracellular uptake [84]. Therefore, PUFAs-based conjugation is a more practical approach for the improvement of endocytosis and cytotoxicity [85].

DOX, one of the important antineoplastic drugs, exerts its broadly cytotoxic effect via topoisomerase II inhibition. Due to its severe systemic toxicities, especially cardiotoxicity and GI disturbances, its application is limited and decreased with other chemotherapeutic agents [86, 87]. Huan et al. synthesized DOX-LNA by coupling the amide bond of DOX to LNA for its cellular uptake investigation in different tumor cells. The intracellular uptake efficiency of DOX-LNA was significantly higher than free DOX, as analyzed by fluorescence imaging and flow cytometry [88]. Furthermore, to compare the potential effects of LNA and palmitic acid (PA) on the endocytosis of DOX, the two fatty acids were attached to DOX through an amide or a hydrazone bond. DOX-LNA presented greater cellular uptake and enhanced cytotoxicity than DOX-PA [89]. It is possibly attributed to the rapid insertion of PUFAs into the biomembrane via inherent tumor-targeting and LNA-mediated endocytosis.

### Enhancing the performance of oral administration

Oral administration is by far one of the most efficient and convenient drug delivery routes of drug intake owing to ease of administration, adjusted dose, excellent patient compliance, and flexibility in the dosing schedule. Unfortunately, the clinical challenges of oral bioavailability are heavily influenced by the physicochemical characteristics of drugs and GI tract irritation [90, 91]. Biological barriers, including the first-pass effect, metabolic instability, and other enzymatic factors, seriously restrict the curative effect of oral delivery. Apart from GI tract obstacles, understanding the limitations of the parent drugs with poor water solubility, low permeability, as well as extreme susceptibility to elimination through efflux transporters are essential steps in the development of prodrugs [92, 93]. Lipinski’s Rule of Five suggests that drug molecules with optimized hydrophilicity/hydrophobicity will achieve desirable oral bioavailability. Among a diversity of prodrug strategies, linking the drug moiety to various lipids has been demonstrated as a promising approach to realize appropriate lipophilicity, thereby improving drug absorption and permeation in oral administration. A key merit of this method is the utilization of the lymphatic transport pathway, in which drug molecules are associated with lipoproteins in the enterocyte process and then transported directly into the blood circulation, thus avoiding undesirable first-pass metabolism and lymphatic leakage [94]. Accordingly, dietary lipids such as triglycerides and phospholipids, absorbed in the intestine via the lymphatic transport pathway, seem to be suitable moieties for drug formulation [95].

CPT and its derivatives are extensively investigated classes of chemotherapeutic agents that act by inhibiting DNA topoisomerase I. As a potent CPT analog, SN38 generates 100 ~ 1000-fold higher antitumor efficacy than irinotecan [96]. However, its oral delivery is extremely impeded by poor solubility, low permeability, and dose-limiting toxicity. In an earlier study, Prestidge’s group designed a series of novel SLPs by esterification with diverse hydrocarbon chain lengths of fatty acids at hydroxyl of SN38. The solubility of SN38-undecanoate, prepared by coupling undecanoic acid to the C_20_ position of SN38, was significantly increased (up to 444-fold) and the cytotoxicity was obviously reduced in comparison to free SN38. Furthermore, the prodrug exhibited improved stability in simulated gastric fluids, preventing its undesirable activation and exposure in the gut. More specifically, in vitro cellular uptake and transmucosal permeability study revealed that SN38 prodrug with optimal chemical modification improved oral absorption as well as promoted transepithelial drug transport [97]. In a recent study by the same group, an optimized self-microemulsifying oral delivery system for SN38 was designed to improve both in vitro/in vivo drug performance. The resulting formulation, purposefully optimized with various lipids, nonionic surfactants, and cosolvents, exhibited maximum drug solubilization under GI conditions, increased transmembrane permeation, and subsequently facilitated oral absorption of drug [98]. Recently, a lipophilic conjugation approach has also been reported in the development of antifungal agents with improved oral bioavailability and metabolic stability [99, 100]. Together, SLPs with an optimal delivery system provide an effective platform for enabling the oral delivery of challenging drugs.

### Increasing the antitumor efficacy and alleviating the systemic toxicity of therapeutic agents

**Fig. 4:**
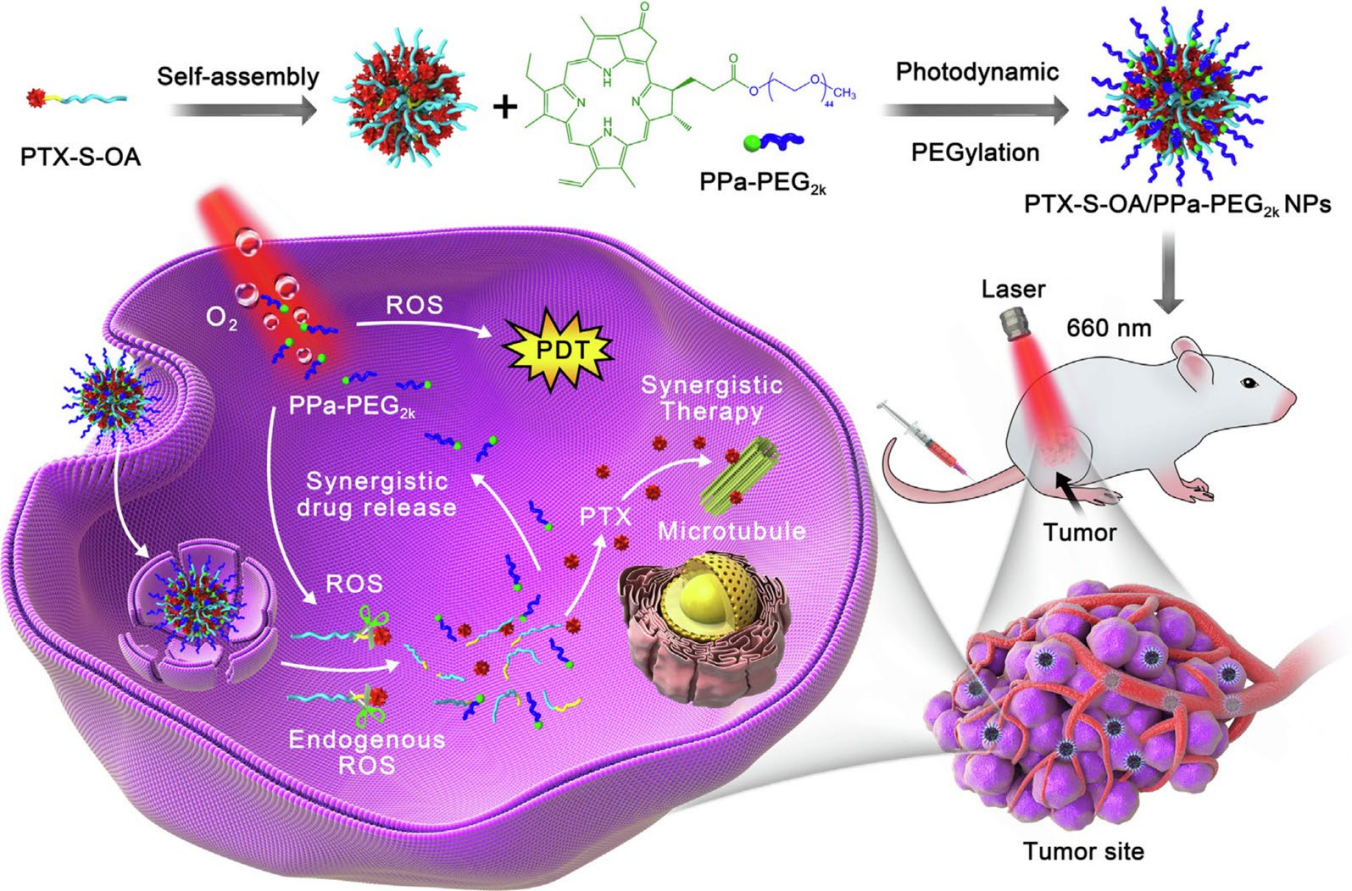
Schematic illustration of prodrug nanoassemblies. The nanoparticles were self-assembled by PTX-S-OA, and PPa-PEG_2k_ was performed for photodynamic PEG coating. Further, PTX-S-OA/PPa-PEG_2k_ NPs were accumulated in the tumors, and self-enhanced core–shell synergistic therapy could be achieved after laser irradiation. (Reprinted from Ref. [102] with permission. Copyright 2019, Elsevier Ltd)

The SLPs-based nanosystem, with the advantages of both nanotechnology and prodrug strategy, emerges as a promising nanoplatform for tumor therapy. As reported, it can achieve high drug loading in DDSs, promote the intracellular uptake and cytotoxicity of chemotherapeutic agents, enhance the performance of orally administered drugs, and therefore realize remarkable therapeutic efficiency in tumor treatment with reduced systemic toxicity (Table 1). As one of the most efficient antineoplastic drugs, PTX is widely used in different types of malignancies. In clinics, the lipidic prodrug PTX-DHA, prepared by attaching PTX to DHA through an ester linkage, revealed greater effects than the free drug at equitoxic doses [101]. Recently, Sun’s group reported a PEG-coated light-activatable prodrug nanoassemblies for synergistic chemo-photodynamic therapy, in which ROS-sensitive oleate prodrug of PTX (OA-S-PTX) was elaborately self-assembled into nanoparticles for chemotherapy (Fig. 4) [102]. In this uniquely engineered prodrug-based DDS, ROS generated by pyropheophorbide a (PPa) not only induced photodynamic therapy (PDT) but also promoted drug release. In comparison with coencapsulation of photosensitizer (PS) and chemotherapeutics into conventional nanocarriers, suffering from inefficient drug loading and aggregation-caused quenching (ACQ) effects, light-sensitive prodrug nanosystem promoted on-demand drug release upon laser irradiation at specific sites and eventually induced prominent tumor ablation.

**Table 1:** SLPs delivery system and their characteristics

Properties	Lipids	Drugs	Refs.
High drug loading	Oleic acid/linoleic acid/stearic acid/phospholipid	Docetaxel/paclitaxel/5-fluorouracil	[72, 80–82]
High intracellular uptake and cytotoxicity	Linoleic acid/palmityl/palmitic acid/linolenic acid	Gemcitabine/doxorubicin	[50, 84, 85, 88, 89]
Enhancing the performance of oral administration	Undecanoate/triglyceride/oleic acid/taurocholic acid/deoxycholic acid	7-Ethyl-10-hydroxycamptothecin/mycophenolic acid/docetaxel/heparin	[95, 97, 109–111]
Increasing the antitumor efficacy with low systemic toxicity	Docosahexaenoic acid/oleic acid/linolenic acid/phospholipid	Paclitaxel/cabazitaxel/porphyrin	[46, 75, 101–103]

**Fig. 5:**
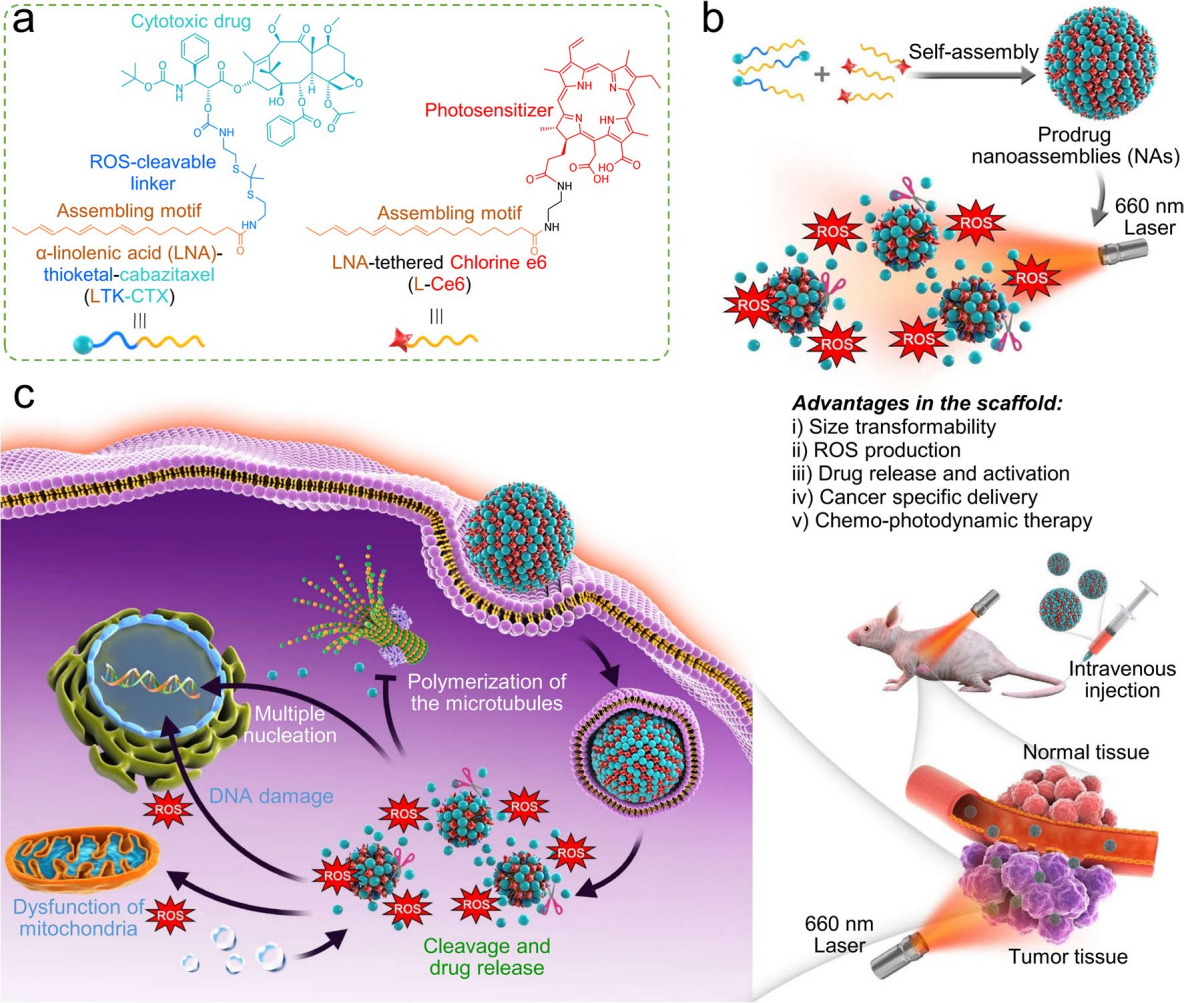
PSPC nanoparticles constructed from SLPs for tumor-specific drug activation and combined chemo-photodynamic therapy. **a** Chemical structures of LTK-CTX and L-Ce6 prodrugs. **b** Self-assembly of the two prodrugs into nanoparticles. Upon light irradiation, the coassembled Ce6 generates ROS and triggers active drug release. **c** Schematic illustrating studies of the in vivo synergistic antitumor efficacy using the PSPC nanoassemblies. (Reprinted from Ref. [103] with permission. Copyright 2020, Elsevier Ltd)

Very recently, the SLPs approach has been developed to reduce surfactant-associated side effects of cabazitaxel (CTX), a semisynthetic product in the taxane family. For example, a series of PUFAs attached to CTX was established by Wang’s research group to fabricate supramolecular nanomedicines (SNM). The results demonstrated that SNM showed higher antitumor efficacy along with improving its safety profile compared to free CTX [46]. Additionally, Huang et al. designed a versatile strategy to prepare a photoactivatable self-assembling prodrug cocktail (PSPC) nanoplatform for light-activated PDT in synchrony with light-triggered on-demand drug activation (Fig. 5) [103]. In the nanosystem, PUFAylated nanoassemblies were composed of CTX prodrug, in which α-linolenic acid was conjugated to CTX via a self-immolative linkage, and LNA-ligated photosensitizer chlorine e6. In multiple tumor models, PSPC nanoparticles exhibited phototoxicity in synchrony with spontaneous chemotherapy for efficient tumor suppression. More importantly, the animals could tolerate the injections of tumor-selective nanotherapy at doses of 50 mg/kg with negligible reduction in body weights. This study unequivocally demonstrated that the self-assembly of SLPs provided a safe and effective delivery strategy for efficient antitumor therapy in the clinic. Additionally, SLPs strategy was also reported in efficient chemo-immunotherapy of colorectal cancer. Nanoparticle with oxaliplatin prodrug and SLPs-based NLG919 reversed the immunosuppressive tumor microenvironment via inactivation the IDO-1. Rationally tailored combination treatment presented synergistic therapeutic efficacy by eliciting effective antitumor immunity with improved survival in vivo [104].

## Biomedical applications of SLPs or SLPs-based novel nanomedicines

An SLPs-based drug delivery system with several advantages, such as enhanced intracellular uptake, high stability in vivo circulation, and improved pharmacokinetics properties, was established to circumvent the delivery obstacles of chemotherapeutic agents. Nowadays, the application of innovative nanotechnology in the medical field presents a promising platform in the development of DDSs. Drug nanocarriers can indeed be considered with specific functionalities to realize precise localization, controlled release, efficient efficacy, ease of administration as well as reduced systemic toxicity. As a promising drug delivery strategy, lipidic prodrugs have been widely applied as building blocks for the fabrication of nanomedicines owing to their physicochemical properties of self-assembly and ability to be incorporated into lipid matrices. It is noteworthy to mention that many of the currently available nanodrugs or those in the late clinical phases are composed of lipids, such as AmBisome® (liposomal formulation of amphotericin B) and Myocet® (liposomal doxorubicin). Accordingly, SLPs or SLPs-based novel nanomedicines have attracted prominent attention in biomedical applications due to their unique properties and have demonstrated significant potential in oral delivery, bioimaging, and therapeutic applications.

### Oral delivery

**Fig. 6:**
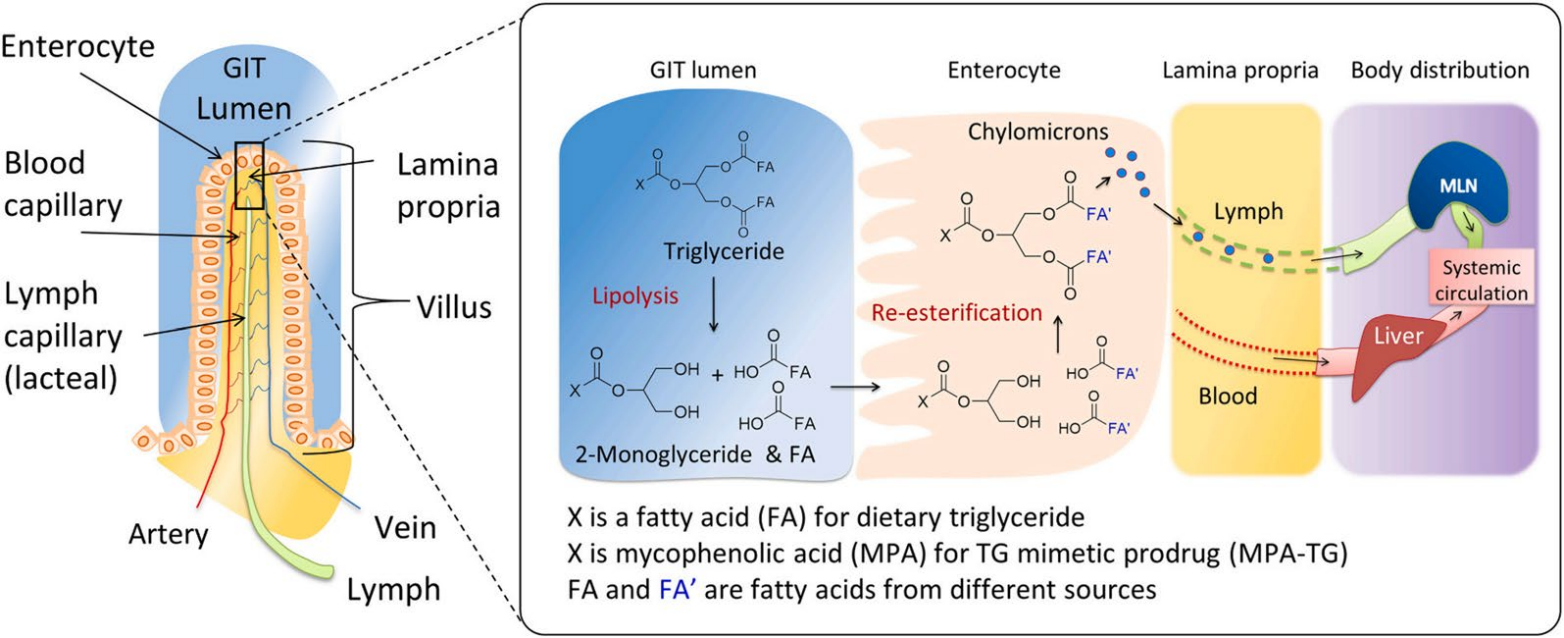
The absorption and transport of TG and TG-mimetic prodrug into the mesenteric lymphatics. In the GIT lumen, TG or TG prodrug is digested by lipases to release 2-monoglyceride and fatty acids, which are absorbed into enterocytes to form new TG derivatives. These TG derivatives are then assembled into lipoproteins and are exocytosed into the underlying lamina propria. (Reprinted from Ref. [107] with permission. Copyright 2021, Elsevier Ltd)

There are various receptors expressed in the epithelial cells for efficient drug absorption via receptor-mediated endocytosis. However, most receptor-mediated strategies are challenged by their efficiency of absorption, targeting ability, or some potential toxicity. To overcome these obstacles, numerous studies pay more and more attention to SLPs or SLPs-based nanoparticles [94]. For instance, Porter’s group designed several lipidic drugs, including mycophenolic acid (MPA), for oral delivery to the mesenteric lymph nodes (MLN) that promote drug integration into the lipid transport pathways [56, 105]. Among them, the most successful approach is a triglyceride (TG)-mimetic prodrug, which has been extensively exploited in oral administration owing to its promising advantages, such as increased oil solubility and drug stability, improved pharmacokinetics, and targeted delivery [58, 106]. In recent studies, the same group exploited the potential pharmacodynamic advantages of the TG-prodrug strategy. In comparison to MPA alone, administration of MPA-TG significantly increased the lymphatic transport of MPA-related species in mice. In addition, under confocal microscopy and flow cytometry analysis, the fluorescent prodrug analog (BODIPY-TG) displayed higher accumulation in MLN and immune cells (Fig. 6) [107].

Additionally, bile acids, which are absorbed in the intestine via the lymphatic transport pathway, are widely used in oral drug delivery systems due to their special physicochemical properties and biological functions [108]. In oral administration, nanotechnology can protect the prodrugs from the harsh conditions in the GI tract, thus realizing desirable therapeutic efficacy by loading different agents. Recently, Khatun et al. reported taurocholic acid-tethered heparin-docetaxel for oral delivery, in which ternary biomolecular conjugates can self-assemble into single nanoparticles (HDTA) with uniform spherical morphology and reproducible function. The oral absorption profile indicated that the drug concentration in plasma was approximately six-fold higher than free heparin, presumably due to the activation of a bile acid transporter in the small intestine. In KB tumor-bearing mice HDTA accumulated only at the tumor site after 24 h of oral delivery, demonstrating its desirable targeting ability. Moreover, antitumor studies both in KB and MDA-MB231 tumor-bearing mice displayed remarkable tumor suppression after treatment with HDTA. The findings suggest a promising approach for efficient antitumor efficacy [109]. Therefore, the rational design of lipidic prodrugs tailored to various medical conditions can create new opportunities for successful oral delivery. Another study developed a strategy to deliver near-infrared (NIR) imaging agents by the oral delivery system, in which low-molecular-weight heparin (LMWH)-bridged deoxycholic acid (LHD) was developed for loading quantum dots via a self-assembly method. After oral administration for bioimaging in SKH1 mice, the fluorescence of Q-LHD was mostly accumulated at the ileum of the small intestine that contained intestinal bile acid transporters. The designed nanocarrier presented safe and efficient merits for oral delivery bioimaging with an admirable absorption and pharmacokinetic profile [110]. Besides, an oleate prodrug of DTX incorporated into a self-nanoemulsifying delivery system was fabricated to promote oral absorption of DTX [111].

### Bioimaging and therapeutic applications

Lipid-based nanoplatforms have been rapidly exploited for biomedical applications in bioimaging and therapy of various diseases and have achieved tremendous clinical success due to their favorable biocompatibility, biodegradation, as well as low immunogenicity. Inspired from the liposomes as nanovesicles [112], numerous studies have paid attention to the lipidic prodrugs as building blocks for the preparation of nanocarriers to deliver therapeutic or imaging agents. In most cases, lipidic prodrugs have the capability to form stable nanostructures via supramolecular self-assembly without the support of additional amphiphilic excipients, which allow them to achieve highly efficient drug loading and prominent efficacy.

**Fig. 7:**
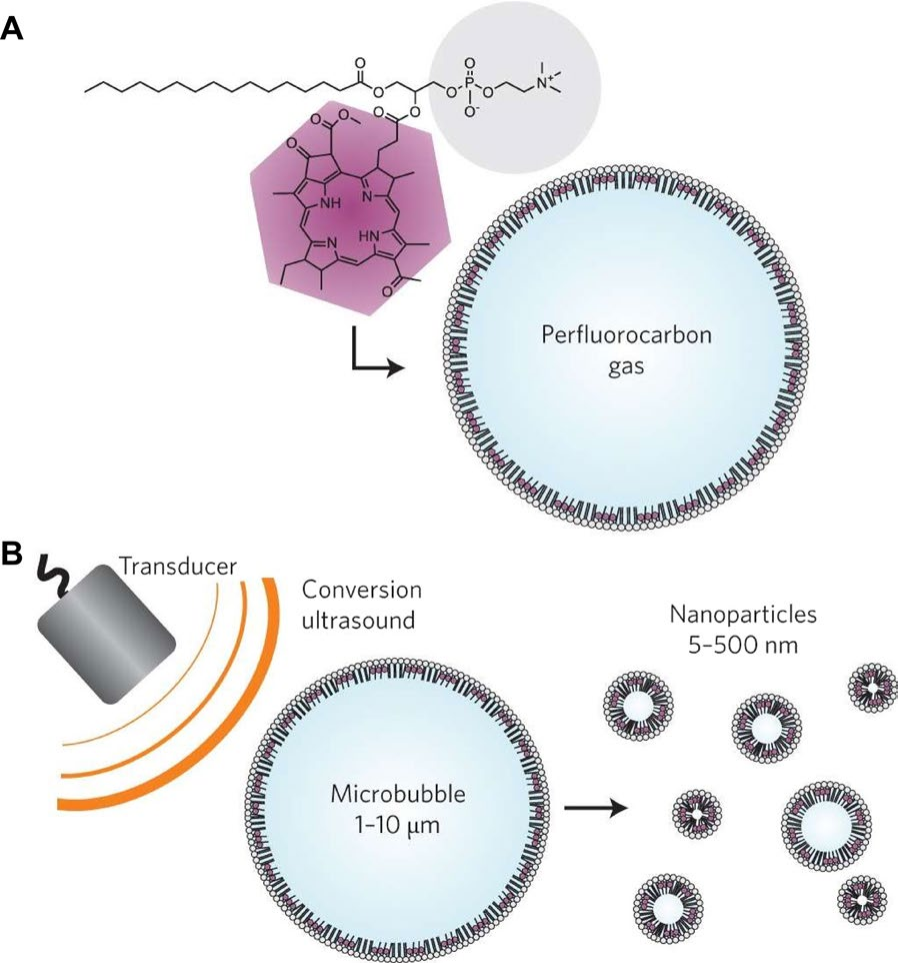
**A** pMBs consist of a porphyrin-lipid shell encapsulating perfluorocarbon gas. **B** Conversion of pMBs to porphysome nanovesicles via sonication with low-frequency. (Reprinted from Ref. [75] with permission. Copyright 2015, Springer Nature Ltd)

By conjugating porphyrins to lipids, Zheng’s group introduced porphysomes nanotechnology, i.e., self-assembly liposome-like nanocarriers with multimodal biophotonic properties, in which high-density porphyrins packing in the lipid bilayer enabled light absorption and conversion to heat with extremely high efficiency, making the porphysome ideal candidates for photoacoustic imaging and photothermal therapy [73, 113, 114]. For instance, Lovell et al. developed an optically active porphysome, in which the porphysome subunits consisted of porphyrin-lipid conjugates generated by an acylation reaction between phospholipid and porphyrin [74]. Similar to inorganic nanoparticles, porphysomes nanovesicles generated tunable extinction coefficients and unique biophotonic properties. Following systemic administration, porphysomes efficiently induced photothermal tumor ablation with minimal acute toxicity in vivo. In addition, metal ions could be incorporated into the lipidic prodrugs-based porphysomes, thus unlocking their potential for magnetic resonance imaging [115]. Recently, porphyrin microbubbles [116–118], porphyrin nanoemulsions [119], porphyrin lipoproteins [76, 120], and nonporphyrin DYEsomes [121] have been gradually reported for phototheranostic applications, thereby expanding the purview of porphysomes nanotechnology. For instance, Huynh et al. designed porphyrin microbubbles for multimodality imaging, in which bacteriochlorophyll-lipid formed a shell around perfluorocarbon gas. On exposure to ultrasound, in situ conversion of microbubbles to smaller nanoparticles could eventually promote ultrasound, photoacoustic, and fluorescent imaging (Fig. 7) [75].

Besides the porphysomes nanotechnology, which benefits from lipidic prodrugs-based nanomedicines, other smart DDSs have been developed for the treatment of various diseases. For example, ursodeoxycholic acid (UDCA)-based dual-functional prodrug nanocarriers were designed by Lee’s research group for bone regeneration through efficient hydrogen peroxide scavenging as well as osteogenic differentiation of mesenchymal stem cells [122]. In another study, Huang et al. designed an SLPs-based nanocarrier for light-activated PDT in synchrony with chemotherapy, which exhibited synergistic activity for efficient tumor suppression in multiple tumor models [103]. Impressively, self-assembly of a thioether-bridged OA-PTX conjugate could realize high drug loading capacity and stimulus-triggered drug release, thus enhancing antitumor activity in a human epidermoid carcinoma xenograft [45]. In addition, the low-density-lipoprotein-inspired nanocarrier provided a prominent approach to the delivery of lipidic prodrugs for efficient cancer treatment. These studies unequivocally demonstrated that the self-assembly of SLPs could provide a valuable and safe strategy for efficient drug delivery and potential translation to clinics.

## Conclusions and perspectives

In this review, we provide an overview of the SLPs strategy and its drug delivery applications. The rational design of SLPs is tailored to specific physiological barriers that facilitate the high-efficiency delivery of chemotherapeutic agents, ultimately enhancing the performance of oral administration and achieving efficient antitumor efficacy with reduced systemic toxicity. At present, numerous lipidic prodrugs or their nanoformulations are under clinical trials, which indicates that the SLPs strategy is a promising approach being utilized in clinical application. Unfortunately, the development of SLPs-based novel nanoparticles for the market is still limited by several factors, especially in the industrial production and scale-up of synthesis and nanoformulations. Moreover, new trends in drug delivery have emerged in recent years, including smart prodrug design responses to different stimuli (e.g., light, temperature, pH, enzymes, and ultrasound) as well as combination therapy. In the future, engineering the more desirable SLPs-based DDSs should comprehensively take specific medical conditions and multifunctional prodrug design into consideration, thus providing great potential for the efficient treatment of various diseases.

## Data Availability

Not applicable.
